# Expanding access to a choice‐based multi‐method PrEP market for HIV prevention

**DOI:** 10.1002/jia2.26512

**Published:** 2025-07-02

**Authors:** Nicolette P. Naidoo, James Ayieko, Virginia A. Fonner

**Affiliations:** ^1^ Wits RHI, University of Witwatersrand Johannesburg South Africa; ^2^ Kenya Medical Research Institute Nairobi Kenya; ^3^ Global Programs and Science, FHI360 Durham North Carolina USA

In the last decade, pre‐exposure prophylaxis (PrEP) has revolutionized HIV prevention. The World Health Organization first recommended daily oral PrEP containing tenofovir as an additional prevention option for all populations at substantial risk of HIV in 2015 [1], then added a recommendation for event‐driven or “on‐demand” PrEP for men in 2019 [2], the monthly dapivirine vaginal ring for women in 2021 [3] and long‐acting injectable cabotegravir (CAB‐LA) in 2022 [4]. More PrEP options are becoming available, such as lenacapavir, which is administered as a sub‐cutaneous injection. Recent clinical trial results suggest lenacapavir injections every 6 months are highly efficacious [5, 6], and early safety and pharmacokinetic data show potential for once‐yearly dosing [7].

Despite the promise of PrEP and the recent proliferation of products, the programmatic rollout of PrEP has been challenging, as system‐level constraints (e.g. cost, policy, operational barriers), social‐level factors (e.g. stigma and lack of normalization of HIV prevention) and individual behaviours (e.g. adherence) have all contributed to limiting overall impact [8, 9, 10]. However, more countries have recently adopted PrEP into national guidelines, and global use of PrEP has increased substantially over the last several years [11]. Research suggests that expanding the menu of PrEP options to better meet the diverse needs and preferences of end users could improve uptake and use [12]. The advent of PrEP methods with different administration routes, discreet formulations and less frequent dosing will potentially enable easier access, more effective use, reduce stigma and, in some cases, allow for the implementation of more flexible delivery channels.

To collate early evidence related to expanding access to a choice‐based HIV prevention market, we invited investigators and research teams across the globe to submit multidisciplinary articles for this supplement, designed to speak to the evaluation and delivery of PrEP choice in diverse settings and for varied populations. After careful consideration, the editorial team selected 15 contributions that illustrate current evidence, implementation learnings and challenges associated with the introduction, uptake and continued use of PrEP within the context of an expanded HIV prevention method‐mix.

Choice in PrEP methods and service delivery approaches have the potential to improve prevention coverage. In a commentary by Schmidt et al. [13], the authors highlight that despite the significant strides made in accelerating oral PrEP scale‐up, it is unlikely that global PrEP targets will be met. In the era of PrEP choice, the authors remind readers that choice is not only about PrEP methods, but also service delivery approaches that have the potential to transform global HIV prevention efforts and maximize prevention coverage. Increased options have been shown to drive demand, allow for user‐centric approaches and facilitate innovation in service delivery models. However, authors caution that it is critical for programmatic challenges, such as PrEP product and service delivery costs, updating monitoring and evaluation and ensuring stakeholder support, to be addressed as there cannot be choice without access.

Resonating with the theme of expanding access through expanding differentiated service delivery options, Kakande et al. [14] report on the post‐trial phase of the landmark SEARCH Dynamic Choice HIV Prevention study conducted in Kenya and Uganda, which offered community‐based choice of oral PrEP/post exposure prophylaxis (PEP) or CAB‐LA. Findings suggest CAB‐LA was feasible and acceptable to deliver with high satisfaction and ease of use reported throughout the study period. Additionally, the authors note that this is likely the first study of its kind to report uptake and experiences of CAB‐LA among heterosexual men in addition to women and demonstrates the demand for long‐acting PrEP among diverse populations.

Several included studies focus on implementing PrEP choice for women. The first two manuscripts speak to offering the monthly dapivirine ring alongside oral PrEP and highlight the importance of offering diverse PrEP options. Fonner et al. [15] present findings on PrEP choice in real‐world settings across five sub‐Saharan African countries as part of the CATALYST study. In their article, they describe uptake and use among individuals offered choice of oral PrEP and the dapivirine ring. This study demonstrates the diversity in choices made in real‐life settings with higher preferences for oral PrEP among their study population. The study further highlights the reasons for choices made including ease of use, efficacy and ease of adherence. This article highlights the continued concern around PrEP continuation and shows the value of presenting choice as a way to enhance retention while highlighting that specific groups, such as adolescent girls and young women (AGYW), still have a greater risk of discontinuing either method, suggesting the need for more support. Hettema et al. [16] report findings from the Eswatini Ring study, which describes method uptake when offered choice, client preferences and experiences with the dapivirine ring, and healthcare provider perspectives on the feasibility and acceptability of offering PrEP choice. Over two‐thirds of participants chose the dapivirine ring, indicating the high acceptability of this vaginal product and demonstrating that PrEP choice is dependent on many factors, including populations offered to and eligible for the products. Additionally, providers conveyed confidence in being able to counsel on PrEP choice but had concerns related to users’ ability to return on time for follow‐up visits and refills.

Dada et al.’s [17] nested qualitative study embedded within an existing implementation science study introducing dapivirine ring alongside oral PrEP in three areas in South Africa explores women's experiences of PrEP choice and factors influencing PrEP choice. The study highlights the importance of provider training, effective counselling tools and tailored communication when offering PrEP choice. Women valued clear, jargon‐free information, visual aids and a welcoming environment, which supported open dialogue. The influence of prior oral PrEP experiences on PrEP choice highlights the need for counselling that addresses specific concerns and preferences.

Wara et al. [18] focus on the preferences and acceptability of long‐acting PrEP in pregnant and lactating people, who face increased vulnerability to HIV. In the PrEPared to Choose study conducted in Cape Town, South Africa among young people 15–29 years of age, the authors report that there was a strong preference for CAB‐LA over oral PrEP. CAB‐LA was found to be highly acceptable, but the authors noted that additional research is needed to evaluate the effect of PrEP choice on continuation among pregnant and lactating people.

Donaldson et al. [19] highlight that increasing uptake of PrEP, including different PrEP options, requires intentional, user‐centred demand generation. The article explores the brand positioning of PrEP using a unified, strategic, evidence‐informed approach that connects with users, specifically how users feel and think about PrEP as a means to prioritize their physical health and mental wellbeing, to live a life uninterrupted by HIV. Their strategy focuses on AGYW and is validated among 18‐ to 24‐year‐old women from Kenya, Zimbabwe and South Africa. The study finds that the process of developing and validating an evidence‐informed strategy for PrEP use confirmed that communication around PrEP should resonate with young women's inner strength and encourage their commitment to use PrEP as an act of self‐love. This is important in enhancing self‐efficacy in the use of PrEP as an HIV prevention option.

Several other studies describe expanding PrEP options among other priority populations, including young people and people with diverse gender and sexual identities. Magno et al. [20] highlight low levels of awareness and high intention to use event‐driven PrEP and long‐acting injectable PrEP among adolescent and young men who have sex with men and adolescent and young transgender women in Brazil. Their article also highlights the diversity in preference when given choice. In designing programmes, these unique differences need to be considered to succeed in averting HIV acquisitions.

Pimenta et al. [21] conducted 120 qualitative interviews with young gender and sexual minority PrEP clients who were participating in the ImPrEP study in Brazil. The findings report reasons why participants chose oral PrEP (less frequent appointments and perceived ease of daily adherence) or CAB‐LA (convenience, practicality and easier adherence). The study also assessed the acceptability of an mHealth intervention to provide information to clients about available PrEP options, and overall, the authors found that the mHealth intervention was perceived as a useful tool to aid in PrEP decision‐making.

Setrakian et al. [22] assessed the relationship of PrEP programme retention with the use of daily oral PrEP, event‐driven PrEP or switching between the two regimens among men who have sex with men in Hanoi, Vietnam. The retrospective analysis of programmatic data spanning several years found that approximately 60% of clients used only daily oral PrEP, about 10% used only event‐driven PrEP and approximately 30% switched regimens. Those who switched regimens had longer median retention in the PrEP programme compared to those who exclusively used daily or event‐driven PrEP, suggesting the importance of allowing and supporting switching between regimens in the context of a multi‐method PrEP market.

In addition to providing perspectives from PrEP clients, one study focused solely on the perspectives of those implementing choice‐based PrEP programmes. Nelson et al. [23] focused on understanding early provider perspectives in EBONI, a phase 4 implementation study of CAB‐LA delivery to Black cis‐ and transgender women in U.S.‐based clinics. In their mixed‐methods study, authors found that providers’ concerns prior to implementation regarding client adherence, insurance verification and client identity decreased following 4 months of implementation. Findings also suggest that clinics successfully identified and used diverse, innovative implementation strategies, such as those that addressed medical mistrust, tracking systems and staff training. Importantly, the study found that addressing population‐specific concerns, educating staff and clients about CAB‐LA, and adjusting clinical flow were key to facilitating implementation.

A viewpoint by Ratevosian et al. [24] describes a policy misalignment in the United States that disincentivizes access to PrEP as insurers bear the cost of providing PrEP because it is currently not covered under the country's Affordable Care Act that would otherwise help compensate insurers for this preventative service. While authors suggest that steps are being taken to ensure that PrEP is covered moving forward, potential changes to HIV prevention funding in the United States could undermine these efforts. Additionally, it is unclear how expanding to a multi‐method market would be handled under these circumstances. This conclusion mirrors that of the Schmidt et al. commentary highlighting that access to PrEP, in all its available forms, is necessary to effect change.

Several articles in the supplement highlight other critical key components to introducing and sustaining the rollout of new PrEP products in the context of a multi‐method market.

Stansfield et al. [25] present results of a comparative modelling analysis on the impact of three different calibrated models of HIV transmission in South Africa over 20 years under multiple scenarios of PrEP expansion with CAB‐LA compared to no PrEP expansion. They conclude that expansion of PrEP coverage would be associated with decreased HIV transmission and that prioritizing PrEP provision and use to populations with high HIV exposure could increase efficiency.

The commentary by Parikh et al. [26] discusses pertinent issues surrounding HIV testing among PrEP users in the context of expanding PrEP options. This article highlights the challenges around currently available tests and possible approaches to resolve ambiguous HIV diagnosis among PrEP users, which is a concern whose magnitude is likely to increase as PrEP use and PrEP options expand.

The viewpoint by Green et al. [27] reflects on the impact of the recent global funding cuts to HIV prevention programmes and the potential challenges with realizing PrEP choice and ultimately prevention coverage. In resource‐constrained environments, now exacerbated by terminated donor funds, countries are urgently required to pivot their response to stabilizing health systems. The authors allude to essential approaches to enable continuity of PrEP access, namely the integration of PrEP services along with investment in public‐private partnerships; institutionalizing differentiated and de‐medicalized PrEP services; simplifying PrEP delivery processes and costs to ensure sustainability; and implementing a number of market‐shaping interventions, including regulatory and procurement support, technology transfer for generic manufacturing and fair pricing.

Collectively, these papers on PrEP choice present emerging evidence from this body of research on how to effectively integrate new PrEP products into routine health systems and expand HIV prevention choice for those who need it most. It further reflects the growing reality that the development and approval of new PrEP methods do not guarantee uptake and effective use. They emphasize that choice is complex and requires countries to consider a number of key aspects.

First, PrEP choice is not just about the methods alone. It is also about access/service delivery, cost, acceptability/feasibility, funding and demand generation (among other factors), as highlighted by many articles in this supplement [13, 19, 23, 24, 25, 27]. PrEP choice does not exist in a vacuum. Expanding PrEP choice also brings new considerations that still require additional investment to understand what is needed for country rollout, such as HIV testing and the ability to detect acute HIV infection in the context of potent long‐acting methods, as raised by Parikh et al. [26].

PrEP preferences are diverse. There is high, varied demand for differing PrEP options across populations, which speaks to the importance of choice in providing options that best fit peoples’ diverse needs and preferences, as reported by Wara et al. [18]. There is a need to consider populations for which full choice might not be available due to population restrictions (young people, pregnant and lactating people, transgender populations) and ensure that policies are changed/expanded once research suggests options are safe/effective for a wider set of populations (such as what happened with the dapivirine ring).

There is demand for new products. Regardless of the options available, people have taken advantage of PrEP choice across all settings. However, availability and uptake within these multi‐method PrEP markets differed across studies. In some studies, oral PrEP and ring were available, in others, it was oral and CAB‐LA, and in one, it was the same formulation (oral PrEP containing tenofovir) but offered as different regimens (daily vs. event‐driven). As noted above, regardless of the menu available, preferences differed. The ability to switch between methods is also an important consideration as this recognizes that client preferences may change throughout the life course.

PrEP choice is feasible to implement across a wide variety of settings and with diverse populations. Several articles shared insights that both end users and providers found it acceptable to deliver choice [16, 17, 23], although data were sparse and may require additional research to fully understand the system‐ and facility‐level requirements to integrate existing and new PrEP methods to achieve efficiency and better client experiences.

As illustrated in this supplement, there is a growing body of evidence demonstrating the early promise of PrEP choice. However, in order to close the research‐to‐implementation gap, it is important that the considerations and insights shared here are utilized by countries to advocate for the availability of multi‐method markets, advance the delivery of choice, optimize HIV prevention efforts and achieve scale of PrEP programmes. Recognizing the uncertainties of funding, it is important now more than ever to advocate for and prioritize HIV prevention and PrEP choice as this remains the most important tool in the arsenal to end HIV.

## COMPETING INTERESTS

The authors declare no competing interests.

## AUTHORS’ CONTRIBUTIONS

VAF, JA and NPN contributed to the initial draft of the manuscript. VAF and JA provided feedback, reviewed and edited the draft. All authors approved the final version prior to submission. NPN finalized and submitted the manuscript.

## FUNDING

Publication of this open‐access supplement was supported by funding from ViiV Healthcare.

## DISCLAIMER

The authors alone are responsible for the views expressed in this issue. They do not necessarily represent the views, decisions or policies of the institutions with which they are affiliated nor any of the funding agencies supporting their work.

## Data Availability

Data sharing is not applicable to this article as no datasets were generated or analysed during the current study.
